# Tobacco consumption and positive mental health: an epidemiological study from a positive psychology perspective

**DOI:** 10.1186/s40359-016-0130-7

**Published:** 2016-05-04

**Authors:** Juan Carlos Bazo-Alvarez, Frank Peralta-Alvarez, Antonio Bernabé-Ortiz, Germán F. Alvarado, J. Jaime Miranda

**Affiliations:** CRONICAS Center of Excellence in Chronic Diseases, Universidad Peruana Cayetano Heredia, Av. Armendáriz 497, Miraflores Lima, Peru; School of Public Health and Administration, Universidad Peruana Cayetano Heredia, Lima, Peru; School of Medicine, Universidad Peruana Cayetano Heredia, Lima, Peru

**Keywords:** Tobacco Consumption, Positive Mental Health, Positive Psychology, GHQ-12, Rural Population, Rural-to-Urban Migrant

## Abstract

**Background:**

Positive mental health (PMH) is much more than the absence of mental illnesses. For example, PMH explains that to be happy or resilient can drive us to live a full life, giving us a perception of well-being and robustness against everyday problems. Moreover, PMH can help people to avoid risky behaviours like tobacco consumption (TC). Our hypothesis was that PMH is negatively associated with TC, and this association differs across rural, urban and migrant populations.

**Methods:**

A cross-sectional study was conducted using the PERU MIGRANT Study’s dataset, including rural population from the Peruvian highlands (*n* = 201), urban population from the capital city Lima (*n* = 199) and migrants who were born in highlands but had to migrated because of terrorism (*n* = 589). We used an adapted version of the 12-item Global Health Questionnaire to measure PMH. The outcome was TC, measured as lifetime and recent TC. Log-Poisson robust regression, performed with a Maximum Likelihood method, was used to estimate crude prevalence ratios (PR) and 95 % confidence intervals (95%CI), adjusted by sex, age, family income and education which were the confounders. The modelling procedure included the use of LR Test, Akaike information criteria (AIC) and Bayesian information criteria (BIC).

**Results:**

Cumulative occurrence of tobacco use (lifetime TC) was 61.7 % in the rural group, 78 % in the urban group and 76.2 % in rural-to-urban migrants. Recent TC was 35.3 % in the rural group, 30.7 % in the urban group and 20.5 % in rural-to-urban migrants. After adjusting for confounders, there was evidence of a negative association between PMH and lifetime TC in the rural group (PR = 0.93; 95%CI: 0.87–0.99), and a positive association between PMH and recent TC in migrants (PR = 1.1; 95%CI: 1.0–1.3).

**Conclusions:**

PMH was negatively associated with TC in rural participants only. Urbans exhibited just a similar trend, while migrants exhibited the opposite one. This evidence represents the first step in the route of knowing the potential of PMH for fighting against TC. For rural populations, this study supplies new information that could support decisions about prevention programmes and psychotherapy for smoking cessation. However, more research in the topic is needed.

**Electronic supplementary material:**

The online version of this article (doi:10.1186/s40359-016-0130-7) contains supplementary material, which is available to authorized users.

## Background

‘It is much better to be wealthy and happy than poor and sick’, a famous quote attributed to Johann Nestroy [24], implicitly suggests the widely held idea that health is merely the opposite of sickness. Although this may be acceptable enough in general medicine, it is certainly not in mental health. Today, we are still trying to expand our understanding of mental health beyond a no-sickness status [24, 45]. Currently, positive mental health (PMH) emerges as an expression of a healthy mind, a balanced emotional life and a strong personality. Happiness, resilience, well-being and optimism – features that are trainable [46] – are some of the features that define PMH in every person. By improving these positive attributes in clients/patients, clinical psychologists and psychiatrists could help to ameliorate some signs and symptoms of common ‘mental disorders’ [30, 46], including tobacco addiction. In other words, clinicians can reinforce their traditional treatment strategies with those from applied positive psychology (the present school of PMH). Moreover, PMH is potentially useful for prevention in healthy people (avoiding relapses). In this study we present preliminary evidence for the potential utility of PMH in preventive clinical practice and epidemiology, by exploring its relationship with tobacco consumption (TC) in naturalistic, non-experimental contexts.

TC is a risky behaviour that represents a concern for public health in low and middle income countries (LMIC), where prevalence of smokers ranks from 16.0 % to 43.3 % [40]. In Peru, reported tobacco users were more severe among rurals (median of 10 cigarettes per month) than among urbans (median of 5.5 per month) or migrants (median of 5 cigarettes per month) [35]. A higher prevalence of tobacco use in rurals has been confirmed in other countries such as India [13] and Mozambique [38]. Furthermore, recent evidence shows how a telephone-based tobacco cessation programme was less effective for rurals than urbans [18]. In sum, TC is a LMIC problem that remarks the inequality between rural and urban populations, claiming mental health studies that can explore alternatives of solutions for both populations.

For positive psychology, the study of the relationship between (positive) mental health and tobacco consumption is an emerging activity, still lacking definitive conclusions. Early evidence showed how cigarette smoking is negatively related to well-being (defined as general satisfaction with own life, including relationships, financial situation, physical and psychological health) [39], and how women who have never smoked had higher levels of well-being than similar ex-smokers and current smokers [15]. Self-efficacy (defined as an individual’s self-perceived ability to cope with stressful or challenging demands, including tobacco or alcohol abstinence) seems to be a strong factor for smoking control in clinical intervention contexts [47]. An increase in resilience (defined as the ability to adapt properly to stressful or extreme situations in life) was accompanied by a reduction in tobacco consumption in high-school students [22]. Optimism (defined as positive perceptions of own life and future) and its relationship with unhealthy habits was studied in 31-year-old men and women, with the results indicating that the proportion of current smokers was higher among pessimists than among optimists [29]. Autonomy (defined as autonomous motivation for initiating and sustaining cessation from smoking, and taking cessation medication) has also been studied as a predictor of smoking cessation while interventions based on self-determination theory have shown their positive effectiveness [49, 50]. In sum, all these studies show evidence of strong and inverse associations between positive mental health indicators and tobacco use.

The mechanisms that explain how people with PMH may be protected against TC can be described as follows. Happiness in these people could be a reflection of their strong personal resources for coping with life; for example, being optimistic about the future or knowing how to face daily difficulties. These people are more protected against depressive episodes and recurrent anxiety [3], both known predictive factors of TC [9]. Resilience is a positive attribute, especially important in critical life situations [25, 42]; it makes a person less likely to relapse into TC. Self-acceptance and self-efficacy are feelings associated with strength of character, independence and a self-supporting personality, which protects against tobacco consumption associated with peer pressure. These attributes are especially important in adolescence, when consumption behaviour has a better prognosis of sustainability [10]. In this situation, PMH can operate as a protective factor against TC, especially for consumers who do not have mental disorders as co-morbidity. Indeed, the first hypothesis that we assessed in our study is “there is an inverse association between PMH and TC”.

In reviewing the literature it is apparent that there is a need for a more integrative measurement of PMH when its relationship with TC is studied. As we have seen above, most researchers have studied different aspects of PMH and its relationship with TC separately. However, people typically have more than one positive attribute behind a unique functioning of PMH, so while one operates the others can have a more discrete action. This circumstance is relevant when the association between PMH and TC is studied: to measure PMH indicators separately can give an incomplete or biased picture of the relationship. It is opportune to remark that PMH has been previously measured [16, 33, 42] and handled [37] like a unique construct, and this is an important aspect to be tapped into by researchers and promoters.

From an epidemiological perspective, it is relevant to know if an association between PMH and TC is generalizable across diverse populations. Psychologists usually affirm that psychological features are culturally bound, as people from different cultures can have different cognitive and behavioural responses to the same stimulus [7]. Since we are interested in obtaining conclusions that are valid inter-culturally, our intention of exploring the relationship between PMH and TC across three important groups in LMIC (rurals, urbans and migrants) is justified. Especially for rurals and migrants there is a lack of information about positive mental health topics. As far as we know, these three populations have shown important differences in terms of traditions, risk behaviours, acculturation, social capital and mental health [31, 51]. Other previous studies have showed that associations between cigarette smoking and some of its known related factors (education and income) differ between non-migrants and rural-to-urban migrants [11], as well as income has a moderation effect on depression that affect cigarette smoking in migrants [12]. Moreover, some positive features such as well-being and self-determination are influenced by the acculturation process of migrants [17]. When this process is not completed, migrants retain particular characteristics that make them different from non-migrants, at least in one of three levels: intrapersonal, interpersonal and citizenship [17]. Considering these evidences, we conclude that an exploration of the association between PMH and TC across these three populations is needed, and differences between them are anticipatable. Indeed, the second hypothesis that we assessed is “the association between PMH and TC differs across rural, urban and migrant populations (the potential effect modifier) because of their psychological and socioeconomic differences”.

To address the gaps identified above, we applied an alternative PMH instrument and compared rural, urban and migrant populations. We have used a general PMH instrument that includes items about happiness, resilience, self-efficacy and self-acceptance to provide a more global perspective of PMH. In addition, we have explored this relationship with regard to three Peruvian populations with known socio-cultural differences: rural non-migrants, urban non-migrants and rural-to-urban migrants [31]. Urban populations are from the coastal areas of Peru and tend to have better economic conditions and access to educational and health services because they live in or near to metropolitan areas. Rural populations include people from the highlands, residing in rural places where poverty and a low quality of educational and health services are common. Migrants are persons who had to migrate from rural settings to the metropolis because of terrorist violence in Peru during the 1980s and 1990s.

In sum, the aim of this investigation is to evaluate the evidence of an association between PMH and tobacco consumption (first hypothesis) and how this association differs across rural, urban and migrant populations (second hypothesis).

## Methods

### Study design

This study is a secondary data analysis using cross-sectional information from the PERU MIGRANT Study. This study was focused on the exploration of differences in cardiovascular risk factors in rural, urban and rural-to-urban migrants in Peru. However, other relevant information was collected, included socio-demographic and mental health outcomes. The questionnaire was administered by trained pollsters, during interviews of 30–40 min. All the questions were done in Spanish, but for non-Spanish speakers a translation was done by pollsters. The aims and methods of this study have already been published and explained in detail [31, 34, 51].

### Participants

Participants were from three populations: non-migrants and residents in the rural zone (*n* = 201), non-migrants and residents in the urban zone (*n* = 199) and rural-to-urban migrants and residents in the urban zone (*n* = 589). The sampling design included stratification by age and sex, where a random selection was applied to every stratum in order to obtain proportional sizes of participants (see Table 1). The inclusion criteria were to be at least 30 years old and the exclusion criteria was not to agree to participate in the study. Each participant in the sample list was visited at home by pollsters. The urban zone was located in Lima, Peru’s capital city. The rural zone was in Ayacucho, a region located in the Peruvian Andes. Migrants were defined as those who moved from Ayacucho to Lima and currently live in Lima. Inclusion and exclusion criteria for this study did not differ from the original study [34].

**Table 1:** Distribution of sex, age, education, income, Positive Mental Health and tobacco consumption by rural, migrant and urban groups in Peru. The PERU MIGRANT study, 2009

		Rural		Migrant		Urban		
		(*N* = 201)		(*N* = 589)		(*N* = 199)		p*
		*n*	(%)	*n*	(%)	*n*	(%)	
Sex								
	Male	95	47.3	280	47.5	92	46.2	0.95
	Female	106	52.7	309	52.5	107	53.8	
Age (years)								
	30-39	61	30.4	154	26.2	54	27.1	0.38
	40-49	55	27.4	178	30.3	51	25.6	
	50-59	48	23.9	173	29.5	61	30.7	
	60-99	37	18.4	82	14.0	33	16.6	
Education								
	without studies	68	33.8	59	10.0	2	1.0	<0.001
	primary	94	46.8	223	37.9	34	17.2	
	secondary	33	16.4	242	41.2	107	54.0	
	superior	6	3.0	64	10.9	55	27.8	
Income								
	<= 160 soles (US$ 50)	109	69.0	8	1.4	2	1.0	<0.001
	between 161–480 soles (US$ 51–150)	32	20.3	143	25.8	36	18.7	
	between 481–800 soles (US$ 151–250)	10	6.3	292	52.6	104	53.9	
	> = 801 soles (> = US$ 251)	7	4.4	112	20.2	51	26.4	
Positive Mental Health								
	(mean(standard deviation))	198	(5.9(1.9))	483	(6.5(1.8))	163	(6.8(1.8))	<0.001
Tobacco Consumption (TC)								
	Lifetime TC	124	61.7	441	76.2	154	78.2	<0.001
	Recent TC	71	35.3	121	20.5	61	30.7	<0.001
	*N*° cigarettes in the last 30 days (median(iqr range))	6	(10(1–20))	37	(5(3–20))	32	(5.5(1–26.5))	0.95

*Chi-square test for categorical variables, ANOVA oneway for positive mental health and Kruskal-Wallis for N° cigarettes in the last 30 days

Lifetime TC: Have you ever smoked a cigarette? Current TC: Are you currently smoker? or Have you smoked in the last six months?

Source: PERU MIGRANT Study dataset

### Variables and conceptual model

In our conceptual model, the primary outcome was tobacco consumption and the main exposure was PMH. We considered sex, age, education and family income as potential confounders. We also considered that being part of a specific population (rural, urban or migrant) may interact with PMH, thereby affecting tobacco consumption as a potential effect modifier.

### Instruments

To assess tobacco consumption (TC), we used two different measures: lifetime TC and recent TC. The question ‘Have you ever smoked a cigarette?’, the lifetime prevalence (cumulative occurrence) question, served to evaluate lifetime TC. This question had three answer choices: 1) yes, 2) yes, but just once to try, and 3) no. The first and second responses were collapsed as one category (yes) of consumption (dichotomic outcome). To assess recent TC, we used cross-referenced information from two questions: 1) When was the last time you smoked? and 2) How many cigarettes have you smoked in the last month? A participant is considered a recent smoker if 1) he/she declared that they smoked in the last six months, or 2) he/she declared that they smoked at least one cigarette in the last month.

PMH was measured by an adaptation of the General Health Questionnaire (GHQ-12), designed and validated previously in two steps (see Additional file 1). The first step included content validation, where items from GHQ-12 were contrasted with items from other tests especially designed for measuring PMH or its more important indicators, such as happiness [2], resilience [41], self-efficacy [43] and self-acceptance [14]. This procedure is supported by the proposal of Joseph and Wood [27], who maintain that positive constructs can be measured by tests originally designed for clinical and psychopathological purposes. A second step consisted of a psychometric revision of reliability and validity using quantitative tools. A procedure with a similar objective was performed by Hu et al. [23], in order to validate GHQ-12 for measuring PMH. After both adaptation steps, we generated a new scale for measuring PMH, maintaining 9 of the original items of GHQ-12. This new scale showed moderate internal consistency (Cronbach’s alpha) globally and for each separate population (global = 0.61, rural = 0.61, migrant = 0.60, urban = 0.68), which are in the acceptable range of 0.60-0.70 for group assessment and group comparisons proposed by Aiken [1]. Exploratory factor analysis showed a one-dimensional solution in every population (see Additional file 1: for a detailed discussion of differences with Hu, and further details about statistical analysis and results).

Sex, age, education and family income variables were measured via the previously-mentioned sociodemographic survey. Age was measured as a continuous variable, although here it has been used in its categorical form (Table 1), given the stratification defined in the sampling design. Education included four levels: no schooling (literate and illiterate), primary education (complete or incomplete), secondary education (high school, complete or incomplete) and superior (undergraduate studies, complete or incomplete). Family income included global income of the participant’s family, including his/her own salary; it is referred to as ‘income’ in the rest of the article.

### Statistical analysis

The first step was to prepare the data for analysis, which included an assessment of the missing values. Next, we conducted an exploratory data analysis, verifying the assumptions of the selected statistical tools. To describe data, we used percentages for categorical variables such as sex, age, education, family income and tobacco consumption (outcome). PMH was treated as a continuous variable and summarised by showing the mean and standard deviation for each population. For bivariate analysis (Table 2), we used simple log-Poisson robust regression models (one model per predictor variable) to estimate prevalence ratios (PR) and a Wald test to obtain *p* values. Multivariate analysis included estimation of two different models. To assess the association between TC and PMH adjusted by confounders (shown also in Table 2 in every population), we estimated this in Model-1:

**Table 2:** Prevalence ratios (Crude and Adjusted) of tobacco consumption (TC) by rural, migrant and urban groups

	Lifetime TC								Recent TC							
	PMH (Crude)				PMH (Adjusted)				PMH (Crude)				PMH (Adjusted)			
	*N*	PR^a^	(CI-95 %)^b^	p*	*N*	PR^a^	(CI-95 %)^b^	p*	*N*	PR^a^	(CI-95 %)^b^	p*	*N*	PR^a^	(CI-95 %)^b^	p*
Rural (N = 201)	98	0.96	(0.91-1.0)	0.12	156	0.93	(0.87-0.99)	0.02	198	0.99	(0.89-1.1)	0.77	156	0.94	(0.83-1.1)	0.33
Migrant (N = 589)	476	1.0	(1.0-1.1)	0.01	448	1.0	(0.97-1.0)	0.96	483	1.2	(1.1-1.4)	<0.01	455	1.1	(1.0-1.3)	0.06
Urban (N = 199)	161	0.99	(0.95-1.0)	0.74	155	0.96	(0.92-1.0)	0.07	163	1.1	(0.90-1.2)	0.54	157	0.98	(0.85-1.1)	0.75

^a^Crude prevalence ratio (PR) has been obtained by a simple log-poisson robust regression model. Adjusted prevalence ratio (PR) has been obtained by the same log-poisson robust regression model, but adjusted by sex, age, education and income. ^b^Confidence Intervals 95 %. *Wald test

*PMH* Positive Mental Health Lifetime TC: Have you ever smoked a cigarette? Recent TC: Are you currently smoker? or Have you smoked in the last six months?

Source: PERU MIGRANT Study dataset

$$ Log(TC)={\beta}_0+\kern0.5em {\beta}_1\kern0.5em PMH+\kern0.5em {\beta}_2 age+\kern0.5em {\beta}_3 sex+\kern0.5em {\beta}_4 education\kern0.5em +\kern0.5em {\beta}_5 income $$

To evaluate interaction between PMH and groups (rural, migrant and urban), we have created a model that includes the interaction variables group*PMH (two dummy variables and one control), henceforth called Model-2:

$$ Log(TC)={\beta}_0+{\beta}_1PMH+{\beta}_2 age+{\beta}_3 sex+{\beta}_4 education+{\beta}_5 income+{\beta}_6 group+{\beta}_7 group\ast PMH $$

To diagnose models, we utilised criteria based on log-likelihood: LR Test, Akaike information criteria (AIC) and Bayesian information criteria (BIC). All PR estimations, crude and adjusted, were performed using a robust log-Poisson regression model [5]. We preferred PRs instead of odds ratios because PRs are more appropriate and easier to interpret in cross-sectional studies when the outcome prevalence is high [21, 44, 48]. A power analysis was performed using a simulation-based approach [32], considering 1000 replications for each specified effect size. This analysis has been included in order to supply relevant information for discussion of non-conclusive results (*p* > 0.05). Throughout, 95 % confidence intervals were calculated. Stata 12.0 for Windows (Stata Corporation, College Station, Texas) was used to perform the analysis.

## Results

### Participant dataset

A total of 989 participants responded to the survey. The final number of analysed cases differs among Tables 1, 2 and 3, given the availability of data (missing complete at random assumption has been verified and pairwise-deletion procedure applied). The highest proportion of missing values was found for income (8.5 %) and PMH (14.9 %). Variable lifetime TC had only 1.3 % of values missing (13 cases).

**Table 3:** Prevalence ratios (Crude and Adjusted) of tobacco consumption (TC) in migrant population by age of migration and time of residence

	Lifetime TC								Recent TC							
	PMH (Crude)				PMH (Adjusted)				PMH (Crude)				PMH (Adjusted)			
	*N*	PR^a^	(CI-95 %)^b^	p*	*N*	PR^a^	(CI-95 %)^b^	p*	*N*	PR^a^	(CI-95 %)^b^	p*	*N*	PR^a^	(CI-95 %)^b^	p*
Age of migration																
0-12 years	163	1.1	(1.0-1.1)	0.04	154	1.0	(0.98-1.1)	0.23	165	1.2	(0.98-1.5)	0.08	**	**	**	**
12-20 years	253	1.0	(0.99-1.1)	0.18	240	0.99	(0.95-1.0)	0.61	258	1.2	(1.0-1.4)	0.01	245	1.1	(0.95-1.3)	0.19
20 or more years	56	1.0	(0.93-1.2)	0.59	50	1.0	(0.85-1.2)	0.95	56	1.2	(0.86-1.8)	0.26	50	1.7	(0.62-4.7)	0.30
Time of residence																
0-20 years	48	1.1	(0.98-1.3)	0.11	47	1.0	(0.90-1.2	0.61	50	1.7	(1.1-2.7)	0.03	49	0.88	(0.46-1.7)	0.69
20-40 years	334	1.0	(1.0-1.1)	0.03	318	1.0	(0.96-1.0)	0.88	339	1.1	(0.97-1.2)	0.15	323	1.0	(0.90-1.2)	0.76
40 or more years	89	1.0	(0.95-1.1)	0.85	79	0.98	(0.91-1.1)	0.56	89	1.9	(1.4-2.5)	<0.001	79	2.1	(1.5-2.9)	<0.001

^a^Crude prevalence ratio (PR) has been obtained by a simple log-poisson robust regression model. Adjusted prevalence ratio (PR) has been obtained by the same log-poisson robust regression model, but adjusted by sex, age, education and income. ^b^Confidence Intervals 95 %. *Wald test. **Model does not converge

PMH: Positive Mental Health Lifetime TC: Have you ever smoked a cigarette? Recent TC: Are you currently smoker? or Have you smoked in the last six months?

Source: PERU MIGRANT Study dataset

### Participant demographics

After revision of the population features (Table 1), distributions for education and income were clearly dissimilar. The rural group mostly had a primary education. However, most urban people had a high-school (secondary) or undergraduate (superior) education. Migrants underwent a position of ‘transition’ between these two groups. Income was similarly distributed with the urban group the richest and the rurals, the poorest. Finally, we detected differences in PMH and tobacco consumption, with the rural group having lower levels of both variables.

### Crude and adjusted association

For crude associations (Table 2), we observed differences in the crude relationship between PMH and tobacco consumption among rurals, migrants and urbans. For example, for migrants there was a positive relationship between PMH and tobacco consumption (both lifetime and recent); however, in rural and urban populations this relationship was negative (at least as a trend). Adjusted results (Model-1 for every group) show a negative association between PMH and tobacco consumption (lifetime) in the rural population: more points on the PMH scale indicate a higher probability of no tobacco consumption (for every unit of increment on the PMH scale, the probability of consuming tobacco is reduced by 7 % across the mean). In fully adjusted models, there was no evidence of a significant association between PHM and tobacco consumption in urban and migrant groups; however, in migrants the trend for positive association deserves attention. Evaluating Model-2 (using lifetime TC), we found an interaction effect among migrant and rural groups (*p* = 0.02, Wald Test) and no interaction effect among migrant and urban groups (*p* = 0.06, Wald Test). However, the global interaction model (Model-2: AIC = 1440; BIC = 1514) was not a better fit than the nested non-interaction model (Model-1: AIC = 1438; BIC = 1502; *p* = 0.43 for the LR test of the nested model with non-robust estimations). Simulation results showed that in the interaction model (Model-2, lifetime TC), the current sample had no more than a 69 % chance of detecting, in urban*PMH interaction, an effect size between −0.04 (PR = 0.96 similar to what was observed in this study for this interaction) and −0.10 (PR = 0.91, bigger than the −0.08 observed in this study for rural*PMH interaction).

### Association and trends in graphics

**Fig 1:**
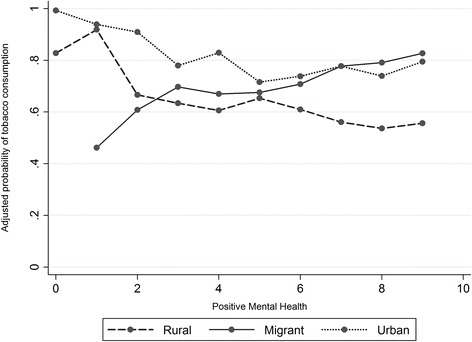
Tobacco consumption and positive mental health for adjusted models by group. Note: the Y-AXIS represents the predicted probability of tobacco consumption, using estimation models adjusted by sex, age, education and income. The X-AXIS corresponds to the direct measurement of the PMH made by our adaptation of the GHQ-12, scaled from 0 to 9

Figure 1 provides a plot of estimated probability of tobacco consumption (Y-Axis) related to direct scaling of PMH (X-Axis), adjusted by sex, age, education and income. The rural curve shows a change from probabilities of tobacco consumption >0.80 at lower points of the PMH scale (0 and 1) to probabilities <0.60 at higher points of the PMH scale (7, 8 or 9). In the urban curve, a similar trend is visible but with a lower magnitude of change: from probabilities of tobacco consumption >0.80 at lower points of the PMH scale (0, 1 and 2) to probabilities <0.80 at higher points of the PMH scale (5, 6, 7 and 8). In migrants, an inverse trend has been observed: from probabilities of tobacco consumption <0.60 at the lowest measured point of the PMH scale (1) to probabilities >0.80 at the highest point of the PMH scale (9).

### Deeper exploration in migrants

In Table 3, attention returns to the trends of positive association between PMH and TC in migrants. A deeper exploration in sub-groups has revealed that migrants who have lived in their new place of residence for 40+ years show a stronger positive association between PMH and recent TC than their counterparts. Stratification by age at migration was also explored, but no relevant results were found.

## Discussion

The results showed above can be summarized in two points: 1) PMH is a protective factor against lifetime tobacco consumption only in the rural population (PR = 0.93, *p* = 0.02); 2) For urban and migrant population we have only detected non-significant and opposite trends: PMH is protective for lifetime TC in urbans (PR = 0.96, *p* = 0.07), but is risky for recent TC in migrants (PR = 1.1, *p* = 0.06). We will discuss these results in the next lines.

PMH is a protective factor against lifetime tobacco consumption only in the rural population (see Table 3). This result has been adjusted by sex, age, education and income which are the main factors associated with TC, considering a previous study in rural population [8]. Free of confounding effect, the relationship between PMH and TC is PR = 0.93, representing an average reduction of 7 % of TC prevalence per every point increased in the PMH scale. This protective association can be explained by a theoretical model where more resilience and happiness can reduce the incidence of anxiety or depressive episodes, both predictive factors of TC. In Peruvian rural population this model has empirical support: they have the highest level of depressive symptoms and tobacco use in the country [31, 35] and our study shows that they have the lowest level of PMH. One adult from rural settings, who lives in poverty and usually depends on agriculture to survive, who has not enough access to the health system and receive just a little support from the Government, is susceptible to fall in critical situations that lead him/her to anxiety or depressive episodes. Those who have developed a strong character for copying the crisis and keep the optimism are covered with a better shield against anxiety and depression. With less incidence of mental illness, these rurals with high PMH can avoid or cease the TC.

For urban and migrant population we have only detected non-significant and opposite trends: PMH is protective for lifetime TC in urbans, but is risky for recent TC in migrants. In urbans there is a similar trend of negative association as in rural people (see Fig. 1 and Table 2), and this trend is visibly different from the positive association trend in migrants (for recent TC). However, the statistical results of Model-2 evaluation have shown that these trends are not enough to conclude a significant difference in the studied association between these populations. Nevertheless, with 69 % of maximum power there remains the possibility of committing a type-II error if we conclude there is no interaction effect for the urban population. Given this uncertainty, it is too hasty to conclude that urban groups and migrants are not intrinsically different. Current trends appear to confirm that migrants (rural-to-urban) and non-migrants (rural and urban) both display distinct associations between PMH and tobacco use. However, new evidence for confirming this difference is needed.

In spite of inconclusive results about differences in the patterns of association between PMH and tobacco in the rural, urban and migrant groups, we believe that the different trend in migrants merits discussion. Peruvian migrants have a history of violence because of terrorism (the principal cause of the Peruvian internal migration phenomenon). In this mass historical migration we recognise an effect on the coastal urban culture (Lima), which gives migrants their particular profile [4]. Major changes suffered by migrants have created a challenging process of adaptation that modified their lifestyle, thinking and behaviour. These extreme requirements of ‘forced adaptation’ (mostly rejected by migrants) have even generated changes in identity that make them a particularly distinct group, alienated from their original culture (rural) and from their new cultural home (urban). This alienation can be expressed through three levels: intrapersonal (related to well-being, self-determination and distress), interpersonal (related to social support), and citizenship (related to sense of belonging, discrimination and stigmatization) [17]. This state of ‘incomplete rural-to-urban cultural transition’ may create a particular psychosocial scenario where positive features (intrapersonal) cannot operate with the same social conditions (interpersonal and citizenship) of non-migrants contexts, altering negative association between PMH and TC that have been detected in rural non-migrants (original culture of these migrants). Actually, this can be the underlying cause of many of the behavioural differences among migrants and urban or rural non-migrants, and may offer the first clue to explaining the differences detected in our study. For example, from the results of Table 3, it is noticeable that migrants living 40 years or more in an urban area (those who are expected to be more ‘acculturated’) still have a positive association between PMH and recent TC (the opposite of what is visible in Table 2 for the native urban population). We recognise the acculturation process is too complex to be analysed and explained properly with only the current information; however, the evidence presented represents a promising beginning.

Some limitations in our study deserve consideration. First, the instrument used for measuring PMH (GHQ-12) was not originally designed for our specific purpose. This problem was offset by a thorough psychometric validation, which included a review of content validity and construct validity (see Additional file 1). Self-report of smoking is another limitation, because it is not the best available measurement of tobacco consumption. However, we believe that the results of this study provide a sufficiently valid approximation (considering that this is a first approach); moreover, ‘lifetime prevalence’ is a known and used variable in the field of addictive behaviour research and its results are easy to compare with others that come from studies on tobacco consumption [6, 19, 26, 28, 36]. Also, we did not control for genes associated with smoking because we did not have this information available; nevertheless, we controlled for other relevant potential confounders. Our cross-sectional design prevents us to make causal inferences; however, it is completely acceptable for doing a first approximation of potential causal relationships. Finally, reflecting on the external validity of the study, we maintain that these results can be formally generalised to the populations that our samples represent, but the same results are also transferable with relative confidence to other groups of Peruvian migrants and non-migrants. Thus, it is clear that, despite the inherent limitations to our research, the information obtained is valuable; although it is not conclusive, it is at least relevant. Given that research into PMH within the field of addiction is still in its infancy, and needs evidence to justify and promote new research, the presentation and dissemination of these results in a timely fashion is important.

We believe that our findings have implications for clinical practice and public health for the rural population in Peru and other similar low and middle income countries (LMIC). Cessation therapies for rural populations can be improved if we consider reinforcing these therapies with positive mental health training. As we have seen, in natural contexts (without systematic training), PMH can work against tobacco consumption as a protective factor. In this sense, complementary PMH training could help to ensure the durability of the positive effects of traditional psychotherapies beyond the clinical space, where psychotherapists cannot monitor and directly influence patient behaviour. Moreover, PMH training can help to develop new preventive initiatives against tobacco consumption at a public health level. Previous studies have shown increasing evidence about how PMH can help, in a large population, to promote general mental health [20]. With our current evidence, we have more support for translating this positive practice to rural populations from LMICs. In sum, our national efforts in the fight against tobacco consumption can be potentiated thanks to PMH promotion and training.

## Conclusion

PMH was negatively associated with TC in rural participants only. Urbans exhibited just a similar trend, while migrants exhibited the opposite one. This evidence represents the first step in the route of knowing the potential of PMH for fighting against TC. For rural populations, this study supplies new information that could support decisions about prevention programmes and psychotherapy for smoking cessation. However, more research in the topic is needed.

### Ethics approval

The protocol for this study was reviewed and approved by the Universidad Peruana Cayetano Heredia’s ethics committee in Peru. The original PERU MIGRANT Study was approved by the same committee, together with the London School of Hygiene and Tropical Medicine.

### Consent for publication

Not applicable

### Availability of data and materials

The dataset supporting the conclusions of this article is available in the Figshare repository via: https://figshare.com/articles/PERU_MIGRANT_Study_Baseline_dataset/3125005

## Additional file

Additional file 1: In this Additional file 1, we present validation procedures and evidence for the adaptation of the General Health Questionnaire (GHQ-12) used in this study for measuring positive mental health (PMH). (DOCX 35 kb)

## Abbreviations

PMH: positive mental health
TC: tobacco consumption
LMIC: low and middle income countries
AIC: akaike information criteria
BIC: bayesian information criteria
PR: prevalence ratio
GHQ-12: general health questionnaire version 12
LR test: likelihood ratio test
