# Supplementary material for: Tobacco consumption and positive mental health: an epidemiological study from a positive psychology perspective
Source: BMC Psychol. 2016 May 4;4:22. doi: 10.1186/s40359-016-0130-7 (PMC4855856; doi:10.1186/s40359-016-0130-7)
Supplement: Additional file 1: — In this Additional file 1, we present validation procedures and evidence for the adaptation of the General Health Questionnaire (GHQ-12) used in this study for measuring positive mental health (PMH). (DOCX 35 kb) [file 40359_2016_130_MOESM1_ESM.docx]

# ELECTRONIC SUPPLEMENTARY MATERIAL

In this supplemental material, we present validation procedures and evidence for the adaptation of the General Health Questionnaire (GHQ-12) used in this study for measuring positive mental health (PMH). As we noted in the main paper, we performed this validation in two steps: content validation and structure validation (both act as evidence of construct validity).

## Step 1: Content validity

Of twelve GHQ items nine were ultimately selected in this phase, after their content validity was considered. To understand the procedure behind this selection, it is necessary to remember that GHQ-12 was originally created as a ‘negative’ measurement of mental health (based on a mental disorders perspective). However, we only selected those items that also provided a feasible assessment of positive aspects of mental health.^[[1]](#footnote-1)^ In other words, some GHQ-12 items are only appropriate as a measurement of mental health from a clinical perspective, but not at all for a positive one, and those items were removed. This is the reasoning that has justified this step, driven by systematic and rigorous content revision.

The first action in step 1 was to contrast items from GHQ-12 against items from other tests especially designed for measuring relevant indicators of PMH. Those tests were: Happiness Scale by Alarcón [1], Resilience Scale by Wagnild and Young [5], General Self-Efficacy Scale by Baessler and Schwarce [6] and Psychological Well-Being Scales by Ryff [2]. The procedure consisted of taking every GHQ-12 item and searching for one or more ‘twin items’ from these PMH tests. In order to conclude that two items are ‘twins’ (one from GQH-12 and another from a PMH test); they must have a very similar meaning even if they do not use exactly the same words. This selection was followed by revision undertaken by three judges (psychologists with experience in applied positive psychology), who checked the previous selection and agreed that nine of the GHQ-12 items were good indicators for measuring PMH (happiness, resilience, self-efficacy and well-being). This information is summarised in Supplemental Table 1.

### *Supplemental Table 1*

Supplemental Table 1. Results of the procedure contrasting GHQ-12 items with items from other tests specifically designed to measure relevant indicators of PMH.

| **GHQ-12 Items** | **Twin item(s) found?** | **PMH test of twin item(s)** | **Twin item(s) -English translation-** | **Judges decision** |
| --- | --- | --- | --- | --- |
| 01. Have you recently been able to concentrate on whatever you’re doing? | no | *** | *** | Item rejected |
| **02. Have you recently lost much sleep due to worry?** | no | *** | *** | Item rejected |
| 03. Have you recently felt that you were playing a useful part in things? | yes | Happiness Scale (Alarcón, 2006) | 14. I feel useless. | Accepted |
| 04. Have you recently felt capable of making decisions about things? | yes | General Self-Efficacy Scale (Baessler & Schwarce, 2000) | 9. If I am in a difficult situation I generally know what I have to do to solve it. | Accepted |
| **05. Have you recently felt under constant strain?** | no | *** | *** | Item rejected |
| **06. Have you recently felt you couldn’t overcome your difficulties?** | yes | Resilience Scale (Wagnild & Young, 2005) | 23. When I'm in a difficult situation, I can usually find my way out of it. | Accepted |
| 07. Have you recently been able to enjoy your normal day-to-day activities? | no | *** | *** | Accepted |
| 08. Have you recently been able to face your problems? | yes | Resilience Scale (Wagnild & Young, 2005) | 1. I usually deal with problems, one way or another. 9. I feel I can handle many things at once. | Accepted |
| **09. Have you recently been feeling unhappy or depressed?** | yes | Happiness Scale (Alarcón, 2006) | 22. I feel sad about who I am. 11. Most of the time I do not feel happy. | Accepted |
| **10. Have you recently been losing confidence in yourself?** | yes | General Self-Efficacy Scale (Baessler & Schwarce, 2000) | 6. When I'm in trouble, I can remain calm because I have the skills to handle difficult situations. 5. Thanks to my own skills and personal resources, I can overcome unforeseen situations. 4. I am confident that I can effectively handle unexpected events. | Accepted |
| **11. Have you recently been thinking of yourself as a worthless person?** | yes | Psychological Well-Being Scales (Díaz et al, 2006) | 7. Usually, I feel proud of who I am and the life I live. 19. I like most aspects of my personality. | Accepted |
| 12. Have you recently been feeling reasonably happy, all things considered? | yes | Happiness Scale (Alarcón, 2006) | 11. Most of the time I do not feel happy. | Accepted |

## Step 2: Structure validity

The second step consisted of a psychometric revision of reliability and validity using quantitative tools. We assessed these properties for the final PMH scale only (i.e. the nine selected items from GHQ-12). First, we re-oriented every item (scaled 0-1 in this version) in order to give them a conventional qualification sense (a higher number means higher PMH). Then, we calculated the mean and standard deviation of every item and the Cronbach´s alpha for the PMH scale, both for the global sample and stratified by populations (see results in Supplemental Table 2).

Additionally, we performed an exploratory factor analysis using the principal factors method on a tetrachoric correlation matrix. For the global sample, the eigenvalue for factor 1 was 2.96, which explains 79% of the variability of the 9 items. Other factors had eigenvalues less than 1 and the scree plot (Supplemental Figure 1) showed a one-factor solution was best. We verified the sphericity assumption (Bartlett Test p<0.001) and sampling adequacy (Kaiser-Meyer-Olkin, Overall KMO=0.71), concluding that it was acceptable. Factor loadings for the one-factor solution are displayed in Supplemental Table 2, both for the overall sample and stratified by populations.

### *Supplemental Table 2*

Supplemental Table 2. Factor loadings in the exploratory factor analysis of the adaptation of the General Health Questionnaire (GHQ-12) for PMH measurement.

|  | Global (n=844, α= 0.61) | | Rural (n=198, α= 0.61) | | Migrant (n=483, α= 0.60) | | Urban (n=163, α= 0.66) | |
| --- | --- | --- | --- | --- | --- | --- | --- | --- |
| GHQ-12 Item* | Mean (sd) | *Factor I* | Mean (sd) | *Factor I* | Mean (sd) | *Factor I* | Mean (sd) | *Factor I* |
| 3. Playing a useful part | 0.91 (0.28) | *0.60* | 0.87 (0.34) | *0.79* | 0.91 (0.28) | *0.40* | 0.97 (0.17) | *0.90* |
| 4. Capable of making decisions | 0.84 (0.37) | *0.60* | 0.86 (0.35) | *0.84* | 0.83 (0.37) | *0.50* | 0.84 (0.37) | *0.56* |
| 6. Can’t overcome difficulties | 0.47 (0.50) | *0.20* | 0.42 (0.49) | *0.16* | 0.49 (0.50) | *0.19* | 0.48 (0.50) | *0.32* |
| 7. Able to enjoy day-to-day activities | 0.80 (0.40) | *0.63* | 0.85 (0.35) | *0.78* | 0.79 (0.41) | *0.60* | 0.80 (0.40) | *0.69* |
| 8. Able to face problems | 0.80 (0.40) | *0.71* | 0.76 (0.43) | *0.79* | 0.81 (0.39) | *0.62* | 0.82 (0.39) | *0.76* |
| 9. Feeling unhappy and depressed | 0.32 (0.47) | *0.53* | 0.30 (0.46) | *0.53* | 0.30 (0.46) | *0.56* | 0.38 (0.49) | *0.81* |
| 10. Losing confidence | 0.63 (0.48) | *0.58* | 0.50 (0.50) | *0.45* | 0.65 (0.48) | *0.70* | 0.70 (0.46) | *0.65* |
| 11. Thinking of self as worthless | 0.73 (0.44) | *0.47* | 0.43 (0.50) | *0.30* | 0.81 (0.39) | *0.60* | 0.86 (0.35) | *0.59* |
| 12. Feeling reasonably happy | 0.87 (0.33) | *0.67* | 0.89 (0.32) | *0.72* | 0.86 (0.35) | *0.67* | 0.90 (0.30) | *0.77* |

* Every item was scaled from 0 to 1.

### *Supplemental Figure 1*

Supplemental Figure 1. Scree plot of eigenvalues after the exploratory factor analysis of the adaptation of the General Health Questionnaire (GHQ-12) for PMH measurement.

## Discussion and conclusions

A procedure such as the one presented in this supplemental material is supported by the proposal of Joseph and Wood [4], who maintained that positive constructs can be measured by tests originally designed for clinical and psychopathological purposes. In theory, we are in a middle position between what they called the weak and strong versions of the ‘continuum approach’. The continuum approach maintains that negative and positive psychological functioning are opposite poles of the same continuum. The ‘weak’ version of this approach (the most popular) postulates that positive and negative functioning are separate dimensions, both related to a higher order called the well-being continuum. The ‘strong’ version of the same approach (the most polemical) maintains the pure vision of the ‘continuum approach’: positive and negative affective states exist – and can be measured – on the same continuum [4]. We say we are in a middle position between both versions because we believe not all positive functions have a negative expression on the same continuum, but it is possible to consider those without a ‘negative pole’ as subsumed by a higher-order continuum (PMH). For example, some effects, such as happiness and sadness, are easier to recognise as opposite expressions, and can also be easily measured as such. However, other positive functions, such as ‘feeling capable of solving problems’ do not have a negative counterpart, so establishing an appropriate measure for these is not easy or free from criticism. However, without detriment to our theoretical position and for current practical purposes, we concluded it is feasible to include both kinds of positive indicators (those with and without negative counterparts) in the same general scale. This is what we have done with our proposed PMH measure: subrogate happiness, resilience, self-efficacy and well-being indicators under one higher-order continuum called positive mental health (taking the weak continuum approach in practice).

This approach for selecting GHQ-12 items is not exactly the same pathway taken by Hu et al. [3]. Their approach was statistically driven, whereas ours has been theoretically driven. This distinction is the main reason there are differences in the final selection of GHQ-12 items; in their study they proposed the use of items 1, 3, 4, 7, 8 and 12 for measuring PMH, while we have used items 3, 4, 6, 7, 8, 9, 10, 11 and 12 for the same objective. We have rejected item 1 because we believe it does not directly measure a positive function (nor do items 2 and 5); at most, they could be considered secondary or indirect expressions of positive functioning (although much more appropriate as direct assessments of a psychopathological symptom). They have rejected items 6, 9, 10 and 11 because these items loaded higher in one factor, which they concluded was a measure of ‘negative’ mental health. Our opposition to this decision is based on the fact that their item selection procedure did not control the natural statistical effect of ‘negatively worded items’, something they also recognise as a limitation. However, we both share the same limitation in our respective studies: we do not have evidence of concurrent validity (using data for correlating the results of proposed GHQ-12 item selection against the results from a test originally written for positive psychological measurements). Nevertheless, step 1 of our validation procedure supports our proposed GHQ-12 item selection because we considered a review of items from PMH tests in our decisions (see Supplemental Table 1).

We concluded that our new GHQ-12 scale for PMH measurement (consisting of the 9 selected items) has sufficient evidence of consistency and validity. As shown in Table 2, this scale indicated moderate internal consistency (Cronbach´s alpha) for the global and individual populations (global=0.61, rural=0.61, migrant=0.60, urban=0.66). Exploratory factor analysis showed a one-dimensional solution in every population. One feature of this solution deserves special mention – the factor loadings of every item vary across populations. This finding is useful for the interpretation of some results of the main paper and we used it in this sense in the Discussion section.

## References

1. Alarcón R. Desarrollo de una Escala Factorial para Medir la Felicidad. R. Interam. Psicol. 2006;40(1):7. Retrieved from: <http://www.redalyc.org/articulo.oa?id=28440110> (accessed 12 April 2015).
2. Díaz D, Rodriguez-Carbajal R, Blanco A, Moreno-Jiménez B, Gallardo I, Valle C, Dierendonck, D. Adaptación española de las escalas de bienestar psicológico de Ryff. Psicothema. 2006;18(3):6.Retrieved from: <http://www.psicothema.com/pdf/3255.pdf> (accessed 12 April 2015).
3. Hu Y, Stewart-Brown S, Twigg L, Weich S. Can the 12-item General Health Questionnaire be used to measure positive mental health? Psychol Med. 2007;37(07):1005-13. DOI: [10.1017/S0033291707009993](http://dx.doi.org/10.1017/S0033291707009993).
4. Joseph S, Wood A. Assessment of positive functioning in clinical psychology: theoretical and practical issues. Clin Psychol Rev. 2010;30(7):830-8. DOI: 10.1016/j.cpr.2010.01.002.
5. Pesce RP, Assis SG, Avanci JQ, Santos NC, Malaquias JV, Carvalhaes R. Cross-cultural adaptation, reliability and validity of the resilience scale. Cad Saúde Pública. 2005;21(2):12. DOI: 10.1590/S0102-311X2005000200010.
6. Sanjuán P, Perez A, Bermúdez, J. Escala de autoeficacia general: datos psicométricos de la adaptación para población española. Psicothema. 2000;12(2):5. Retrieved from: <http://www.psicothema.com/pdf/615.pdf> (accessed 12 April 2015).

1. These ‘negative or positive’ meanings, which directly referred to psychological functioning, should not be confused with those that referred to positive or negatively worded item orientation. [↑](#footnote-ref-1)
